# Irisin ameliorates UUO-induced renal interstitial fibrosis through TGF-β1/periostin/MMP-2 signaling pathway

**DOI:** 10.1371/journal.pone.0299389

**Published:** 2024-06-13

**Authors:** Yashu Wang, Xinna Deng, Jinying Wei, Zhaohua Yang, Yunxia Du, Shan Song, Yonghong Shi, Haijiang Wu

**Affiliations:** 1 Department of Pathology, Hebei Medical University, Shijiazhuang, China; 2 Department of Oncology, Hebei General Hospital, Shijiazhuang, China; National Institutes of Health, UNITED STATES

## Abstract

Renal fibrosis is the most common pathway in progressive kidney diseases. The unilateral ureteral obstruction (UUO) model is used to induce progressive renal fibrosis. We evaluated the effects of irisin on renal interstitial fibrosis in UUO mice. The GSE121190, GSE36496, GSE42303, and GSE96101 datasets were downloaded from the Gene Expression Omnibus (GEO) database. In total, 656 differentially expressed genes (DEGs) were identified in normal and UUO mouse renal samples. Periostin and matrix metalloproteinase-2 (MMP-2) were selected to evaluate the effect of irisin on renal fibrosis in UUO mice. In UUO mice, irisin ameliorated renal function, decreased the expression of periostin and MMP-2, and attenuated epithelial-mesenchymal transition and extracellular matrix deposition in renal tissues. In HK-2 cells, irisin treatment markedly attenuated TGF-β1-induced expression of periostin and MMP-2. Irisin treatment also inhibited TGF-β1-induced epithelial-mesenchymal transition, extracellular matrix formation, and inflammatory responses. These protective effects of irisin were abolished by the overexpression of periostin and MMP-2. In summary, irisin treatment can improve UUO-induced renal interstitial fibrosis through the TGF-β1/periostin/MMP-2 signaling pathway, suggesting that irisin may be used for the treatment of renal interstitial fibrosis.

## 1. Introduction

Chronic kidney disease (CKD) is a major disease that threatens public health. Renal tubulointerstitial fibrosis is a key factor in CKD progression. The histopathological features of renal interstitial fibrosis include deposition of extracellular matrix (ECM) components, including collagen 1 and III, loss of renal tubular cells, accumulation of fibroblasts, and sparseness of the peritubular microvascular [1]. Epithelial-mesenchymal transition (EMT) is a key mechanism involved in the progression of renal interstitial fibrosis and is characterized by renal tubular epithelial cells acquiring a mesenchymal phenotype and myofibroblast function [2]. Unilateral ureteral obstruction (UUO) causes a range of pathophysiological and morphological changes in the renal parenchyma, including interstitial fibrosis, inflammation, and apoptosis of renal tubular cells [3]. It is extremely important to understand the underlying mechanism of renal injury and interstitial fibrosis in the UUO model.

Irisin was discovered in 2012 as a muscle secretion hormone generated by the breakdown of fibronectin type III domain-containing protein 5 (FNDC5) under exercise stimulation [4]. Irisin can enhance glucose and fat metabolism by combining with fat cells to convert white fat into brown fat [5]. Previous studies have shown that irisin reduces fibrosis of the liver, pancreas, heart, and skeletal muscles, and plays a protective role in these organs [6–9]. Recent studies have shown that dojuksan, an herbal decoction that can increase the plasma concentration of irisin, can block the progression of renal fibrosis by increasing circulating irisin [10]. These results also indicated that irisin has a potential therapeutic effect on the progression of renal fibrosis.

In the present study, we aimed to investigate the anti-fibrotic effect of irisin in UUO mice and TGF-β1-induced HK-2 cells and to explore its potential molecular mechanism. We first performed a bioinformatics analysis based on Gene Expression Omnibus (GEO) database to identify differentially expressed genes (DEGs) between sham-operated mice and UUO mouse kidney samples. Periostin and matrix metalloproteinase-2 (MMP-2) have been identified as two important hub genes. We then evaluated the renoprotective effects of irisin via the TGF-β1/periostin/MMP-2 pathway in UUO mouse and TGF-β1 stimulated HK-2 cells through a series of experiments. This study provides new insights into the precise treatment of CKD.

## 2. Materials and methods

### 2.1 Bioinformatics analysis

Microarray gene expression profile chips GSE121190 [11], GSE36496 [12], GSE42303 [13], and GSE96101 [14] were used for bioinformatics analysis. We matched the probe IDs of the four datasets with the gene symbols on the platform and deleted probe IDs that matched no or multiple gene symbols. If multiple IDs corresponded to a single gene, the highest expression value of these probes was defined for that gene. The limma package of the R software program (version 4.1.2, https://www.r-project.org/) was used to normalize and filter DEGs in each dataset. A correction p value < 0.05 and |logFC| ≥ 1 were selected as the cutoff criteria for DEGs. These DEGs were used for subsequent analysis.

To explore the biological functions and pathways of these DEGs, Gene Ontology (GO) and Kyoto Encyclopedia of Genes and Genomes (KEGG) enrichment analysis were performed using the DAVID database (https://david.ncifcrf.gov/). We then downloaded the enrichment analysis results for subsequent analyses. Statistical significance was set at P <0.05.

Protein-protein interaction (PPI) networks were constructed using the STRING database (https://www.string-db.org/), and the interaction results were downloaded for subsequent analysis. The Cytoscape software (http://www.cytoscape.org/) was used to visualize the PPI network. The CytoHubba plug-in in the Cytoscape software was used to identify the top 20 hub genes in the DEGs. The Molecular Complex Detection (MCODE) plug-in in the Cytoscape software was used to select meaningful modules.

### 2.2 Materials

Antibodies against periostin (19899-1-AP), MMP-2 (10373-2-AP), collagen 1 (14695-1-AP), E-cadherin (20874-1-AP), α-SMA (14395-1-AP), and TNF-α (17590-1-AP) were purchased from Proteintech (Chicago, IL, USA). Antibodies against fibronectin (ab2413) were purchased from Abcam (Cambridge, UK). Periostin pcDNA, MMP-2 pcDNA, and control empty plasmids were obtained from GenePharma (Shanghai, China). Recombinant human TGF-β1 was purchased from PeproTech (Rockville, MD). Recombinant irisin was purchased from Sigma-Aldrich (St. Louis, MO, USA). The plasmid overexpressing *Fndc5* (NM-153756) was purchased from OriGene Technologies, and an empty plasmid vehicle was obtained from Beijing Zoman Biotechnology Co., Ltd. All the culture media were purchased from Gibco-BRL (Grand Island, NY, USA). The polyvinylidene difluoride (PVDF) membranes were purchased from Millipore (Billerica, MA, USA).

### 2.3 Animals and groups

Male C57BL/6JN mice aged 6–8 weeks were acquired from Beijing HFK Biotechnology Company. The Experimental Animal Center at Hebei Medical University provided free food and water. Animal Experiment Ethics Review Committee of Hebei Medical University (IACUC-Hebmu-2023006) gave its approval to the work’s animal experimentation plan. The mice were divided into four groups at random: sham group (sham operated mice, n = 6), UUO group (UUO mice, n = 6), UUO+vehicle group (UUO mice treated with empty plasmid vehicle, n = 6) and UUO+irisin group (UUO mice treated with overexpressed *Fndc5* plasmid injection group, n = 6). After intraperitoneal injection of 1% pentobarbital sodium, each mouse was anesthetized and placed in the right lateral position. Skin was prepared locally and disinfected strictly with iodophor. An incision was made about 0.5 cm below the left rib on the back, and the kidney and ureter were freed. The upper and lower thirds of the ureter were ligated with 4–0 silk thread, cut from the middle of the ligation, and then the muscle and skin were sutured continuously. Surgical manipulation without ureteral ligation was performed on the sham group. To guarantee the therapeutic effect of irisin, *Fndc5* expression plasmid (25 μg, dose 1 mg/kg) was injected into the kidney through the tail vein one day before surgery and 3, 7, and 11 days later. A empty plasmid was also added in the same way. After 14 days, blood was taken from the heart puncture, and then the animals were deeply euthanized with pentobarbital sodium (150–300 mg/kg, intraperitoneal injection) to alleviate suffering. After 15 minutes of high-speed centrifugation at 3000 rpm at 4°C, the plasma was separated, and the serum was stored at -80°C for subsequent monitoring of serum creatinine and total cholesterol (Nanjing Jiancheng Biotechnology Co., Ltd). According to the manufacturer’s instructions, the concentration of Irisin (Elabscience, Wuhan, China) in serum was determined using an ELISA kit. In addition, the left kidney was stripped of the renal capsule under a low temperature environment, half of which was fixed in paraformaldehyde for histological monitoring, and the other half was used for Western blotting and reverse transcription-polymerase chain reaction (RT-PCR).

### 2.4 Cell culture

HK-2 cells were purchased from the American Center for Type Culture Collection and cultured in DMEM-F12 medium containing 10% fetal bovine serum, 100 U/m L penicillin and 100 μg/m L streptomycin at 37°C in a 5% CO2 incubator. HK-2 cells were cultured in basal medium, 10 ng/mL TGF-β1, TGF-β1 + 15 ng/mL irisin for 48 hours. According to the manufacturer’s packaging recommendations, HK-2 cells were stably transfected with Periostin overexpression plasmid, MMP-2 overexpression plasmid or empty plasmid vector using Lipofectamine 2000 (Invitrogen, Carlsbad, CA) after the cell fusion in the basal medium reached 40–50%. After 6 hours of culture in the medium without 1% serum, the basic medium was replaced and TGF-β1 and irisin were added to continue the culture for 48 hours. Each experiment was repeated at least 3 times.

### 2.5 Protein extraction and western blotting

Total proteins (30–50 μg) were extracted from kidney tissue and HK-2 cells using RIPA buffer (Solarbio, Beijing, China) containing a protease-phosphatase inhibitor mixture. After lysis on ice for 30 min, cell lysates were centrifuged at 4°C, 12000 rpm for 30 min. Protein concentrations were quantified using a BCA Protein Concentration Assay Kit (Solarbio, Beijing, China). Protein samples were separated by SDS-PAGE and transferred to PVDF membrane (Burlington Millipore, MA, USA). The membrane was blocked with 5% skimmed milk powder at 37°C for 1 h. The cells were incubated overnight with anti-periostin (1:1000), anti-MMP-2 (1:1000), anti-fibronectin (1:500), anti-collagen 1 (1:1000), anti-E-cadherin (1:1000), anti-α-SMA (1:1000), and anti-TNF-α (1:500) antibodies. The band intensity was analyzed using the ImageJ software (National Institutes of Health).

### 2.6 Histology and immunohistochemistry

Briefly, all the samples were fixed in 4% paraformaldehyde and embedded in paraffin. Paraffin sections were cut into 2 μm sections for hematoxylin-eosin (HE), periodic acid-Schiff (PAS), and Masson’s trichrome staining. For immunohistochemical analysis, paraffin sections were cut into 4 μm, dewaxed with xylene, and rehydrated with gradient ethanol. Internal peroxides were inactivated using 3% hydrogen peroxide in 100% methanol for 30 min. Antigen retrieval was performed in 10 mM citric acid buffer in a microwave oven for 10 min. Next, 10% PBS normal goat serum was added to the slices and incubated at room temperature for 30 min to block nonspecific antibody binding. The sections were incubated overnight at 4°C with primary antibodies against periostin, MMP-2, fibronectin, collagen 1, E-cadherin, α-SMA, and TNF-α. After rinsing in PBS, the sections were incubated with biotinylated secondary antibodies and streptavidin labeled with horseradish peroxidase. After staining with diaminobenzidine to produce brown tissue, the sections were counterstained with hematoxylin.

### 2.7 RNA extraction and quantitative RT-qPCR analysis

Total RNA was isolated and extracted using the TRIzol reagent (Invitrogen Life Technologies, Carlsbad, USA). HiScript III RT SuperMix (Vazyme Biotech, Nanjing, China) was used to synthesize cDNA, according to the manufacturer ’s instructions. The primers were as follows: *FN* (fibronectin), forward:5’- acaagcatgtctctctgcca-3’ and reverse:5’- ccagggtgatgcttggagaa-3’; *COL1A1* (collagen 1), forward:5’- ccgtgacctcaagatgtgc -3’ and reverse:5’- cttgaggttgccagtctgc -3’; *E-cadherin*, forward:5’- aggccaagcagcagtacatt -3’ and reverse:5’- gggggcttcattcacatcca -3’; *α-SMA*, forward:5’- ataacatcaagcccaaatc -3’ and reverse:5’- acttcccaaagcatcagc -3’; *TNF-α*, forward:5’- gagtgacaagcctgtagccca -3’ and reverse:5’- gcaatgatcccaaagtagacc -3’; *IL-6*, forward:5’- aaagaggcactggcagaaa -3’ and reverse:5’- tttcaccaggcaagtctcct -3’; *β–catenin*, forward:5’-ggctcttgtgcgtactgtccttc-3’ and reverse:5’- cttggtgtcggctggtcagatg -3’. Real-time PCR was performed in a 96-well photoreaction plate using ChamQ Universal SYBR qPCR Master Mix (Vazyme Biotech, Nanjing, China). Real-time PCR feedback was conducted on an Agilent Mx3000P QPCR System (Agilent, CA, USA). The fold-change in gene expression was calculated using the 2^−ΔΔCT^ method.

### 2.8 Immunofluorescence staining

HK-2 cells were fixed in 4% paraformaldehyde on a six-well slides for 30 min. The cells were then incubated with specific primary antibodies against periostin, MMP-2, fibronectin, collagen 1, α-SMA, E-cadherin, TNF-α, and IL-6 overnight at 4°C. Subsequently, HK-2 cells were exposed to IFKine TMgreen-labeled goat anti-rabbit IgG at 37°C for immunofluorescence staining, or IFKine TMgreen-labeled goat anti-rabbit IgG and IFKine TMred-labeled goat anti-mouse IgG for double immunofluorescence staining.

For the tissues, the kidneys were embedded with the O.C.T compound (optimal cutting temperature) in Sakura Finetek (Torrance, CA), and their slices were prepared. Subsequently, the sections were air-dried and fixed in ice-cold acetone for 20 min, and then blocked with normal goat serum (10%) at 37°C for 30 min. Primary antibodies against periostin and MMP-2 were incubated overnight at 4°C. Finally, the kidney tissues were incubated with IFKineTM green-conjugated goat anti-rabbit IgG and IFKineTM red-conjugated goat anti-mouse IgG at 37°C for 2 h, and the nuclei were stained with DAPI. Images were obtained using an Olympus microscope.

### 2.9 Determination of serum creatinine and total cholesterol in mice

Serum creatinine (C011-2-1) and total cholesterol (A111-1-1) detection kits were purchased from Nanjing Jiancheng Biotechnology Co., Ltd. According to the manufacturer’s instructions, the serum creatinine and total cholesterol levels of mice were detected by creatine oxidase method and COD-PAP method, respectively.

### 2.10 Statistical analysis

Data are expressed as means ± standard deviation (SD). Statistical analysis was performed by one-way analysis of variance (ANOVA) using SPSS. Statistical significance was defined as p<0.05.

## 3. Results

### 3.1 Identification of periostin and MMP-2 as DEGs between Control and UUO Mice

Four microarray gene expression profiles, GSE121190

(https://www.ncbi.nlm.nih.gov/geo/query/acc.cgi?acc=GSE121190), GSE36496

(https://www.ncbi.nlm.nih.gov/geo/query/acc.cgi?acc=GSE36496), GSE42303

(https://www.ncbi.nlm.nih.gov/geo/query/acc.cgi?acc=GSE42303), and GSE96101

(https://www.ncbi.nlm.nih.gov/geo/query/acc.cgi?acc=GSE96101), were downloaded from the GEO database (https://www.ncbi.nlm.nih.gov/geo/). The platform for GSE121190, GSE36496, GSE42303 and GSE96101 are GPL11180 [HT_MG-430_PM] Affymetrix HT MG-430 PM Array Plate, GPL4134 Agilent-014868 Whole Mouse Genome Microarray 4×44K G4122F (Feature Number version), GPL8321 [Mouse430A_2] Affymetrix Mouse Genome 430A 2.0 Array and GPL4134 Agilent-014868 Whole Mouse Genome Microarray 4×44K G4122F (Feature Number version), respectively. We selected 19 UUO-subjected C57BL/6 mouse kidney samples and 15 sham-operated C57BL/6 mouse kidney samples for bioinformatic analysis.

**Fig 1:**
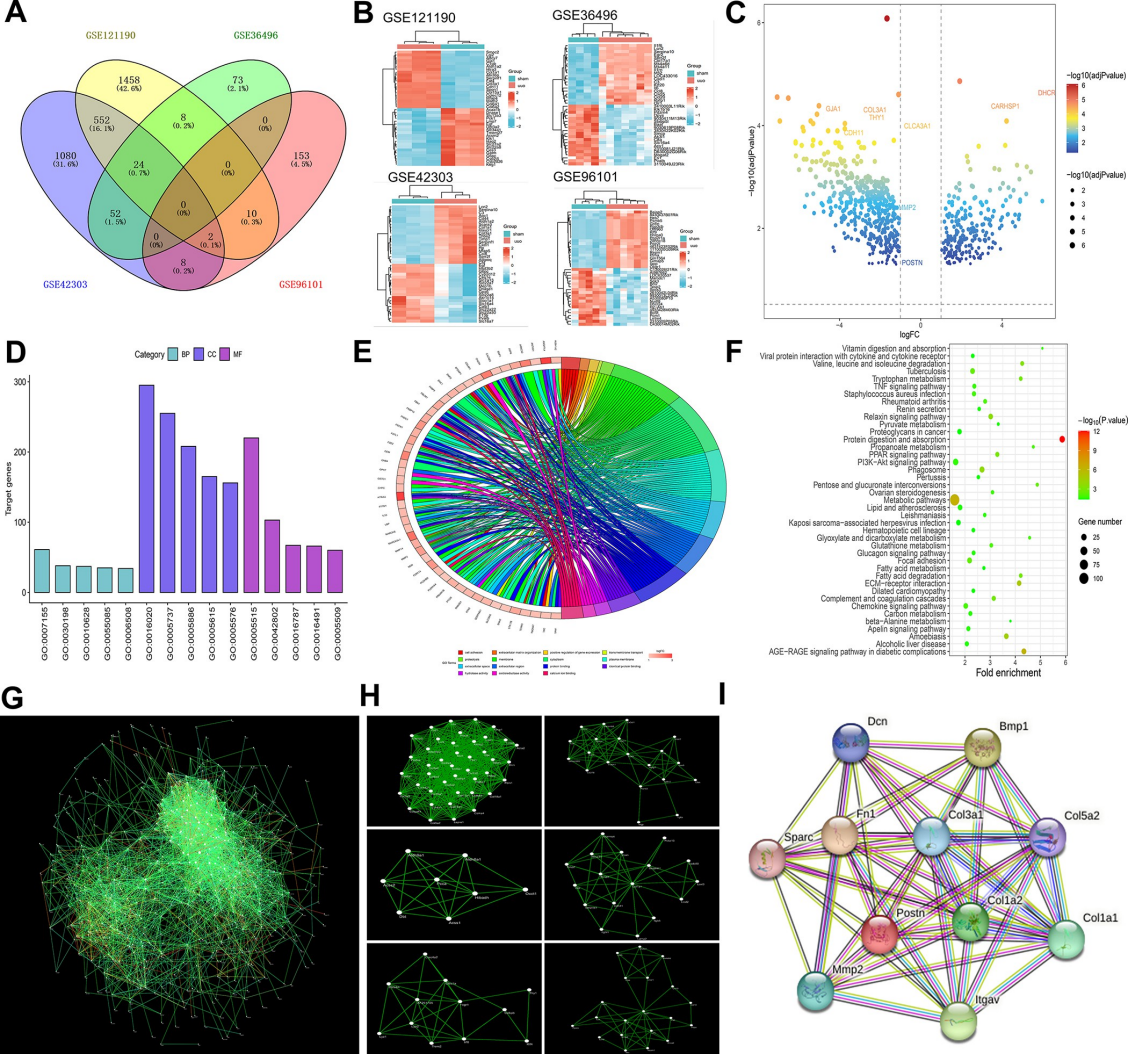
Identification of DEGs and biological pathways between Control and UUO Mice Kidney Tissues in GSE121190, GSE36496, GSE42303 and GSE96101. **A** Venn diagram analysis of four GSE. **B** Heatmap of 656 DEGs. Red represented the up-regulated gene, blue represented the down-regulated gene. The legend on the top right represented the logarithmic fold change of the genes. **C** Volcano plot of 656 DEGs. **D** Top 15 enriched GO terms of 656 DEGs. **E** The distribution of 656 DEGs in different GO enrichment functions. **F** KEGG pathway enrichment analysis of 656 DEGs. **G** PPI network of 656 DEGs. Every node represents a gene-encoded protein. Every edge represents the interaction between them, and the edge color represents the core degree of the protein. **H** Top 6 functional modules were identified from the PPI network. **I** Gene network diagram of interaction with periostin from the STRING database.

According to the criterion of adjusted p value <0.05, and |logFC| ≥1, 2054, 157, 1718, and 173 DEGs were identified in GSE121190, GSE36496, GSE42303, and GSE96101, respectively. We then created a Venn diagram (**Fig 1A**) using the website (http://bioinfogp.cnb.csic.es/tools/venny/index.html). In total, 656 DEGs were screened from the intersection of two or more datasets. These DEGs were visualized using a clustering heat map (**Fig 1B**) and volcano map (**Fig 1C**). GO functional enrichment analysis of DEGs was performed according to biological process (BP), cellular component (CC), and molecular function (MF). The top five enriched GO terms for BP, CC, and MF of the 656 DEGs are shown in **Fig 1D and 1E**. The GO analysis details of the DEGs are presented in **Table 1**. KEGG pathway enrichment analysis showed that the DEGs were enriched in 41 pathways (**Fig 1F**). The PPI network of DEGs was constructed using the Cytoscape software, as shown in **Fig 1G**. We used Cytoscape’s MCODE plugin to identify the six most important functional modules of the PPI network (**Fig 1H**). The CytoHubba plug-in of Cytoscape was used to identify the top 20 hub genes from the PPI network according to their degree values and LogFC values (**Table 2**). Among the top 20 hub genes, periostin (postn) had the largest absolute value of logFC. We then screened for proteins that interacted with periostin using the STRING database. As shown in **Fig 1I**, we found that MMP-2 significantly interacted with periostin, which is also one of the top 20 hub genes. Therefore, the periostin/MMP-2 pathway was selected for further investigation.

**Table 1:** Detailed GO analysis of 656 DEGs.

Category	Term	Count	*P* value
BP	cell adhesion	61	7.66E-16
BP	positive regulation of transcription from RNA polymerase II promoter	48	0.069211678
BP	extracellular matrix organization	38	3.61E-20
BP	positive regulation of gene expression	37	6.69E-05
BP	transmembrane transport	35	5.70E-07
CC	membrane	295	4.00E-14
CC	cytoplasm	255	0.001874472
CC	integral component of membrane	226	0.099581369
CC	plasma membrane	208	0.001485001
CC	extracellular space	165	1.20E-34
MF	protein binding	220	5.11E-05
MF	metal ion binding	130	0.092658893
MF	identical protein binding	103	2.26E-06
MF	hydrolase activity	67	0.034806376
MF	oxidoreductase activity	66	5.62E-17

**Table 2:** The degree values of the top 20 hub genes in 656 DEGs.

Name	Gene title	Score	LogFC
*Periostin*	Postn, osteoblast specific factor	75	-6.6593
*Col6a1*	collagen, type XVI, alpha 1	71	-5.854694
*Lox*	lysyl oxidase	77	-5.80715
*Timp1*	tissue inhibitor of metalloproteinase 1	68	-5.598883
*Col1a1*	collagen, type I, alpha 1	98	-5.41496
*Fbn1*	fibrillin 1	66	-5.248234
*Cd44*	CD44 antigen	78	-4.8648
*Dcn*	decorin	64	-4.740977
*Fn1*	fibronectin 1	140	-4.71892
*Col1a2*	collagen, type I, alpha 2	81	-4.68327
*Col3a1*	collagen, type III, alpha 1	77	-4.24626
*Col5a2*	collagen, type V, alpha 2	63	-4.03243
*Mmp*	matrix metallopeptidase 2	59	-3.881839
*Col5a1*	collagen, type XV, alpha 1	70	-3.793624
*Acta2*	actin, alpha 2, smooth muscle, aorta	90	-3.73478
*Sparc*	secreted acidic cysteine rich glycoprotein	63	-2.930213
*Itgam*	integrin alpha M	72	-2.83717
*Bgn*	biglycan	64	-1.844696
*Stat3*	signal transducer and activator of transcription 3	67	-1.052972
*Egf*	epidermal growth factor	85	4.851208

### 3.2 Irisin suppressed periostin/MMP-2 expression in TGF-β1-stimulated HK-2 cells

**Fig 2:**
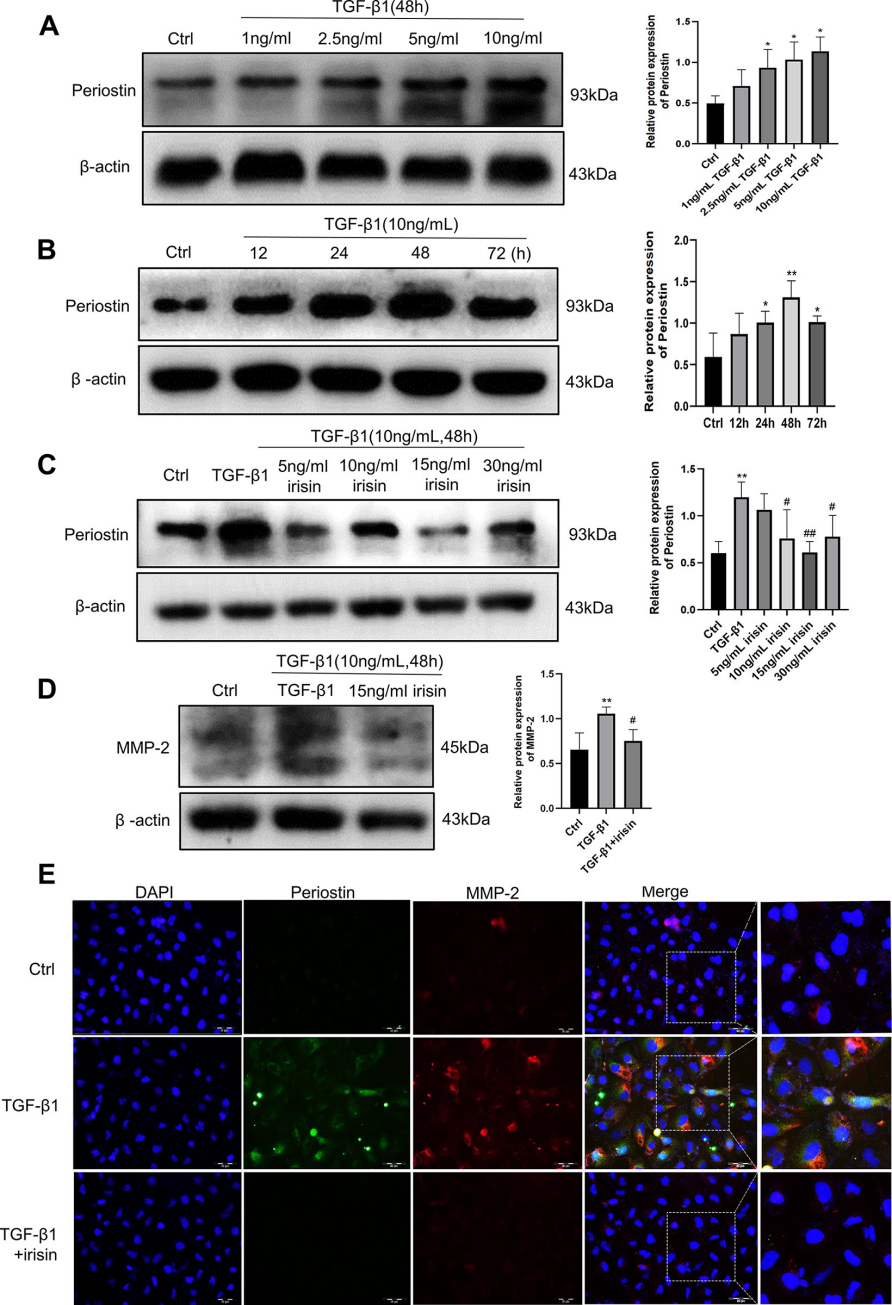
TGF-β1 induced periostin expression and activity in HK-2 cells, and 15ng/mL irisin inhibited periostin and MMP-2 expression stimulated by 10 ng/mL TGF-β1. **A** Expression of periostin stimulated by 0, 1, 2.5, 5 and 10 ng/mL TGF-β1 for 48 hours. **B** The time-response effects of TGF-β1 (10 ng/mL) on periostin. **C** Effects of 5, 10, 15 and 30 ng/mL irisin on the expression of periostin in HK-2 cells stimulated by TGF-β (10 ng/mL) analyzed by western blotting. **D** Effects of 15 ng/mL irisin on the protein levels of MMP-2 in HK-2 cells stimulated by TGF-β (10 ng/mL) analyzed by western blotting. **E** The representative immunofluorescence images of periostin (green) and MMP-2 (red) in HK-2 cells. All experiments were performed independently at least in triplicate. Values are expressed as means ± SD. *p<0.05 versus control group. **p<0.01 versus control group. #*p*<0.05 versus TGF-β1 group. ##*p*<0.01 versus TGF-β1 group.

To determine the interaction between periostin and TGF-β1, we explored periostin expression in TGF-β1-stimulated HK-2 cells *in vitro*. As shown in **Fig 2A**, TGF-β1 stimulation for 48 hours resulted in a dose-dependent increase in periostin expression in HK-2 cells. We then stimulated HK-2 cells with 10 ng/mL TGF-β1 to observe periostin protein expression at different time points. As shown in **Fig 2B**, the results showed that the protein expression of periostin peaked at 48 hours.

We further explored the effect of recombinant irisin treatment on periostin and MMP-2 expression in HK-2 cells exposed to 10 ng/mL TGF-β1. The results showed that compared with the control group, TGF-β1 stimulation of HK-2 cells significantly increased the expression of periostin. In contrast, recombinant irisin treatment attenuated this trend induced by TGF-β1. The effect of irisin at 15 ng/mL was the most significant (**Fig 2C**). Similarly, the expression of MMP-2 was also elevated in the TGF-β1 group compared to that in the control group, and this effect was significantly suppressed by 15 ng/mL irisin (**Fig 2D**). Moreover, we performed double immunofluorescence staining and found that periostin and MMP-2 co-localized in TGF-β1-stimulated HK-2 cells (**Fig 2E**).

### 3.3 Effect of irisin on TGF-β1-induced ECM secretion, EMT and inflammation in HK-2 cells

**Fig 3:**
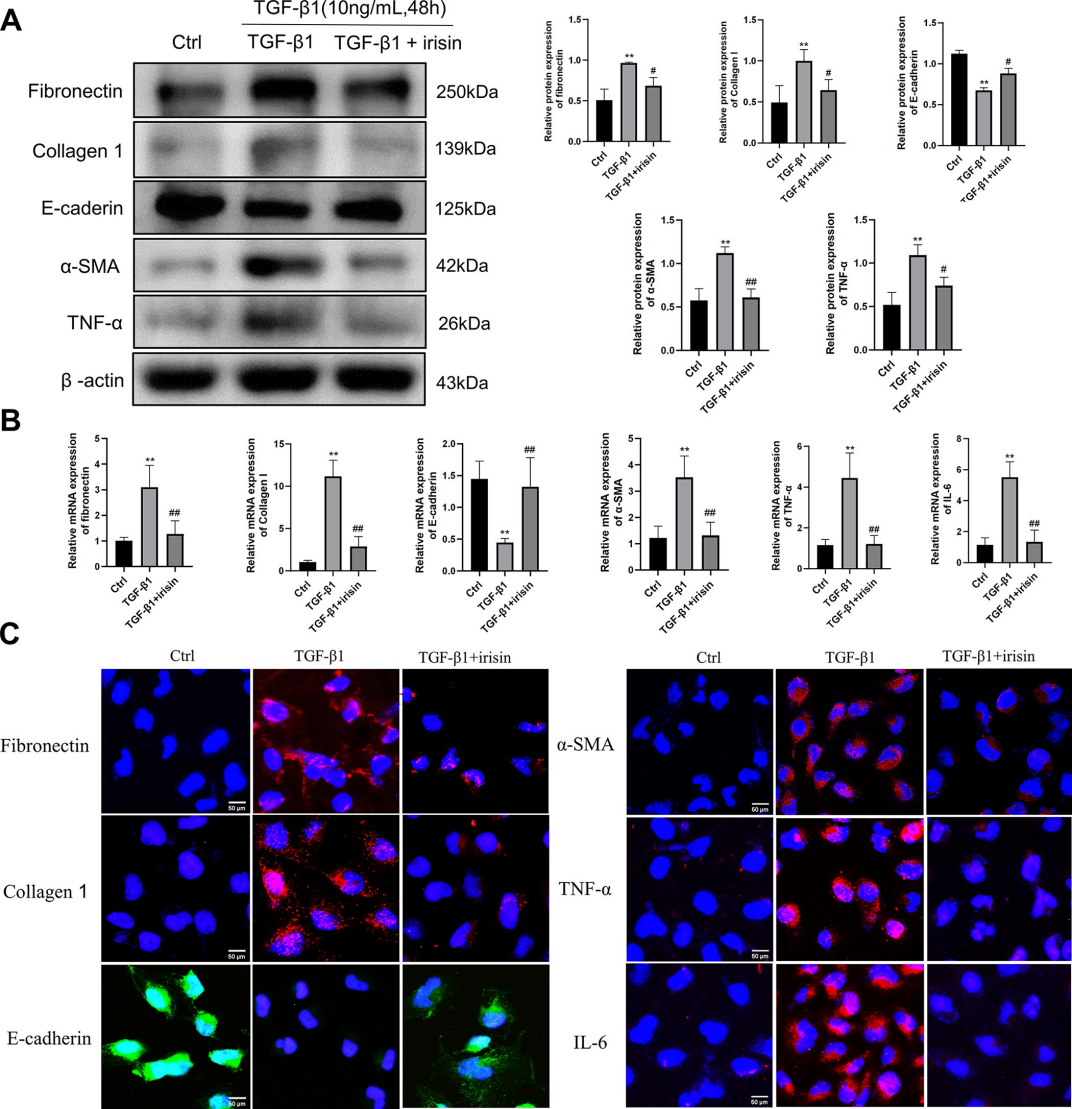
Irisin inhibited TGF-β1-induced ECM, EMT and inflammatory responses in HK-2 cells. **A** A representative western blotting and relevant quantification of fibronectin, collagen 1, α-SMA, E-cadherin and TNF-α in HK-2 cells. **B** RT-PCR was used to ascertain the mRNA levels of fibronectin, collagen 1, α-SMA, E-cadherin, TNF-α and IL-6. **C** Representative immunofluorescence images of fibronectin, collagen 1, α-SMA, E-cadherin, TNF-α and IL-6. All experiments were performed independently at least in triplicate. Values are expressed as means ± SD. *p<0.05 versus control group. **p<0.01 versus control group. #*p*<0.05 versus TGF-β1 group. ##*p*<0.01 versus TGF-β1 group.

To elucidate the role of irisin in TGF-β1-induced fibrosis and inflammation in HK-2 cells, we evaluated the expression of fibrosis- and inflammation-associated proteins in HK-2 cells. Western blotting results showed that compared with the control group, the protein levels of ECM-related indicators (collagen 1 and fibronectin), EMT-related indicators (α-SMA), and inflammatory indicators (TNF-α) were increased, whereas the protein expression of E-cadherin was decreased in HK-2 cells stimulated with 10 ng/mL TGF-β1. Irisin treatment significantly reversed these changes (**Fig 3A**). Moreover, we performed RT-PCR (**Fig 3B**) and immunofluorescence staining (**Fig 3C**) experiments and found that the results were consistent with those of western blotting.

### 3.4 Overexpression of periostin/MMP-2 attenuated the effects of irisin on TGF-β1-induced EMT, ECM deposition and inflammation response in HK-2 cells

**Fig 4:**
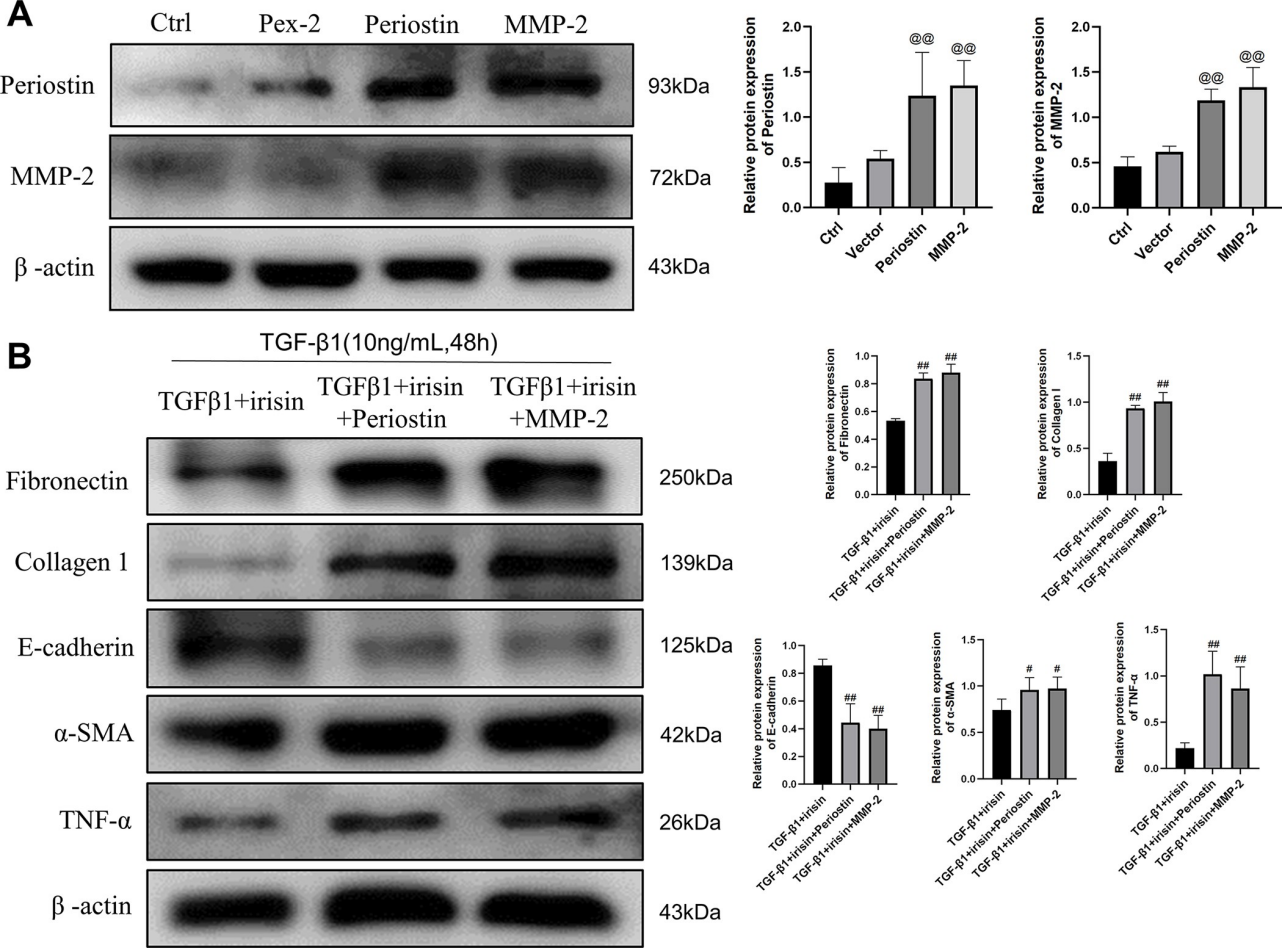
Overexpression of periostin and MMP-2 can significantly reduce the anti-fibrosis and anti-inflammatory ability of irisin. **A** A representative western blotting and relevant quantification of periostin and MMP-2 in HK-2 cells. **B** Representative western blotting and relevant quantification of fibronectin, collagen 1, α-SMA, E-cadherin and TNF-α in HK-2 cells. All experiments were performed independently at least in triplicate. Values are expressed as means ± SD. @ *p*<0.05 versus pex-2. @@ *p*<0.01 versus pex-2. #*p*<0.05 versus TGF-β1+irisin group. ##*p*<0.01 versus TGF-β1+irisin group.

To further elucidate the molecular mechanism of irisin on TGF-β1-induced fibrosis and inflammation, we evaluated the effects of irisin on EMT, ECM production, and inflammatory responses by overexpressing periostin and MMP-2 in HK-2 cells stimulated by TGF-β1. HK-2 cells were transfected with a periostin overexpression plasmid, MMP-2 overexpression plasmid, or pex-2 empty plasmid vector. As shown in **Fig 4A**, compared with the pex-2 empty plasmid vector, the periostin and MMP-2 overexpression plasmids significantly increased the protein levels of periostin and MMP-2 in HK-2 cells. We further elucidated whether irisin could inhibit TGF-β1-induced fibrosis and inflammation in HK-2 cells after the overexpression of periostin and MMP-2. As shown in **Fig 3A**, the protein expression of fibronectin, collagen 1, α-SMA, and TNF-α was markedly decreased by irisin, whereas E-cadherin levels were markedly increased by irisin in TGF-β-stimulated HK-2 cells. However, these irisin-induced changes induced by irisin treatment were significantly attenuated by periostin and MMP-2 overexpression (**Fig 4B**).

### 3.5 Irisin treatment alleviated kidney damage in UUO mice

**Fig 5:**
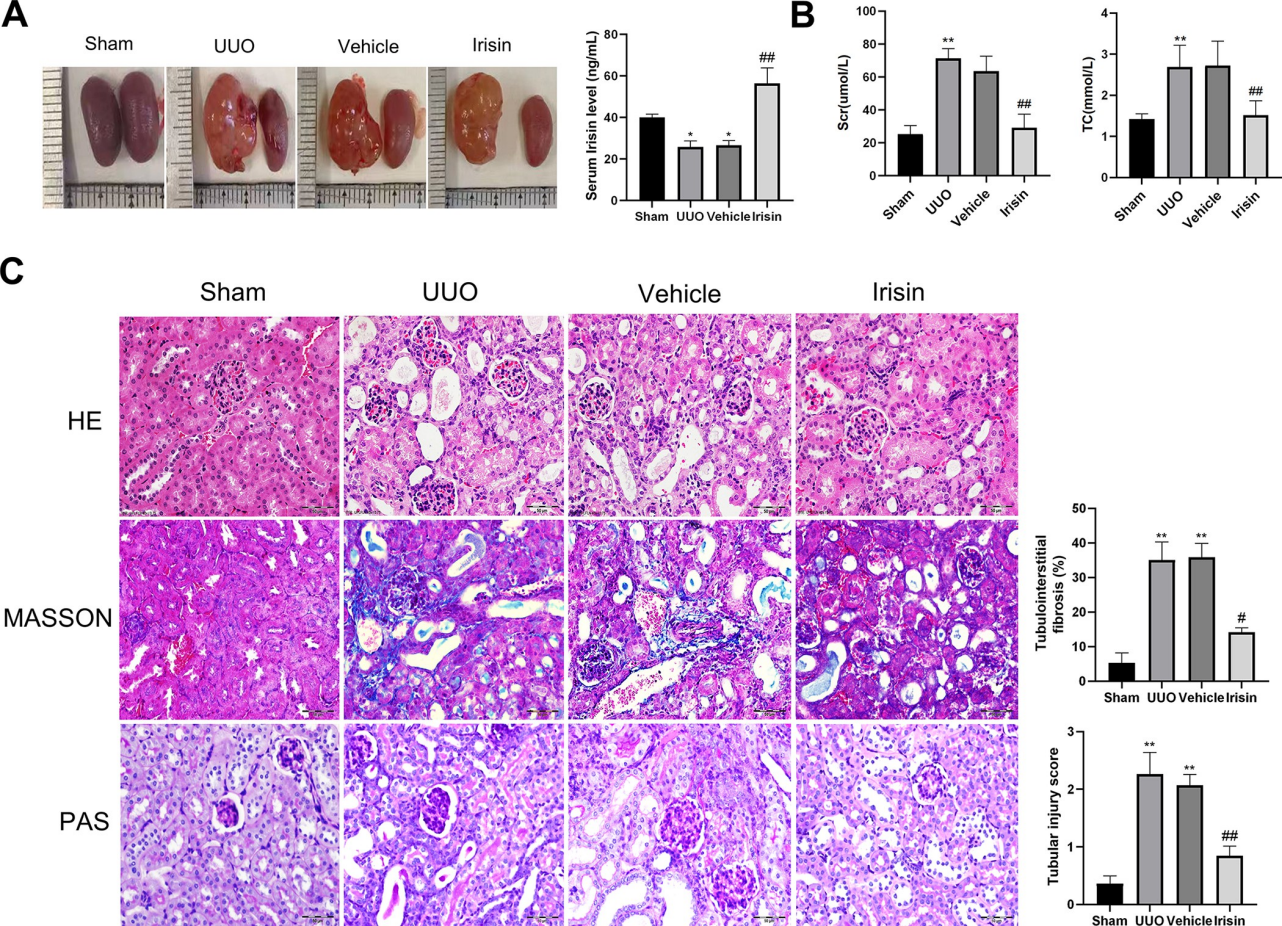
Irisin improved the changes of renal function indexes and renal morphology in UUO mice. **A** Representative photos of kidneys and serum irisin levels were detected by ELISA. **B** Detection of biochemical indexes including serum creatinine (Scr) and total cholesterol (TC) in each group. **C** Representative HE, Masson and PAS staining images of renal tissue from four groups of mice. All experiments were performed independently at least in triplicate. Values are expressed as means ± SD. *p<0.05 versus Sham group. **p<0.01 versus Sham group. #*p*<0.05 versus Vehicle group. ##*p*<0.01 versus Vehicle group.

To confirm the protective effects of irisin *in vivo*, UUO mice were treated with the plasmid overexpressing the *Fndc5* gene, which increased irisin concentration in the blood, for two weeks. **Fig 5A** displays representative images of the kidneys of C57BL/6 mouse. The levels of serum creatinine (Scr) and total cholesterol (TC) levels of the UUO mice significantly increased, indicating a significant amount of renal impairment, and the irisin treatment group significantly alleviated this phenomenon (**Fig 5B**). Then, we explored the role of irisin treatment on histopathological changes in the kidneys. HE staining showed that, compared with the sham group, the renal tissues from the UUO group mice displayed significant renal tubular injury, including renal tubular dilatation, atrophy, and epithelial cell proliferation. Meanwhile, PAS staining showed that the renal tissues from the UUO group had obvious glycogen deposition after 14 days. However, these characteristics were significantly alleviated in the renal tissues of the irisin-treated group. Masson’s trichrome staining and quantitative evaluation confirmed that UUO mice treated with irisin exhibited a significant reduction in renal fibrosis compared to the kidneys of UUO mice (**Fig 5C**).

### 3.6 Irisin treatment alleviated the expression of periostin and MMP-2 in the UUO kidneys

**Fig 6:**
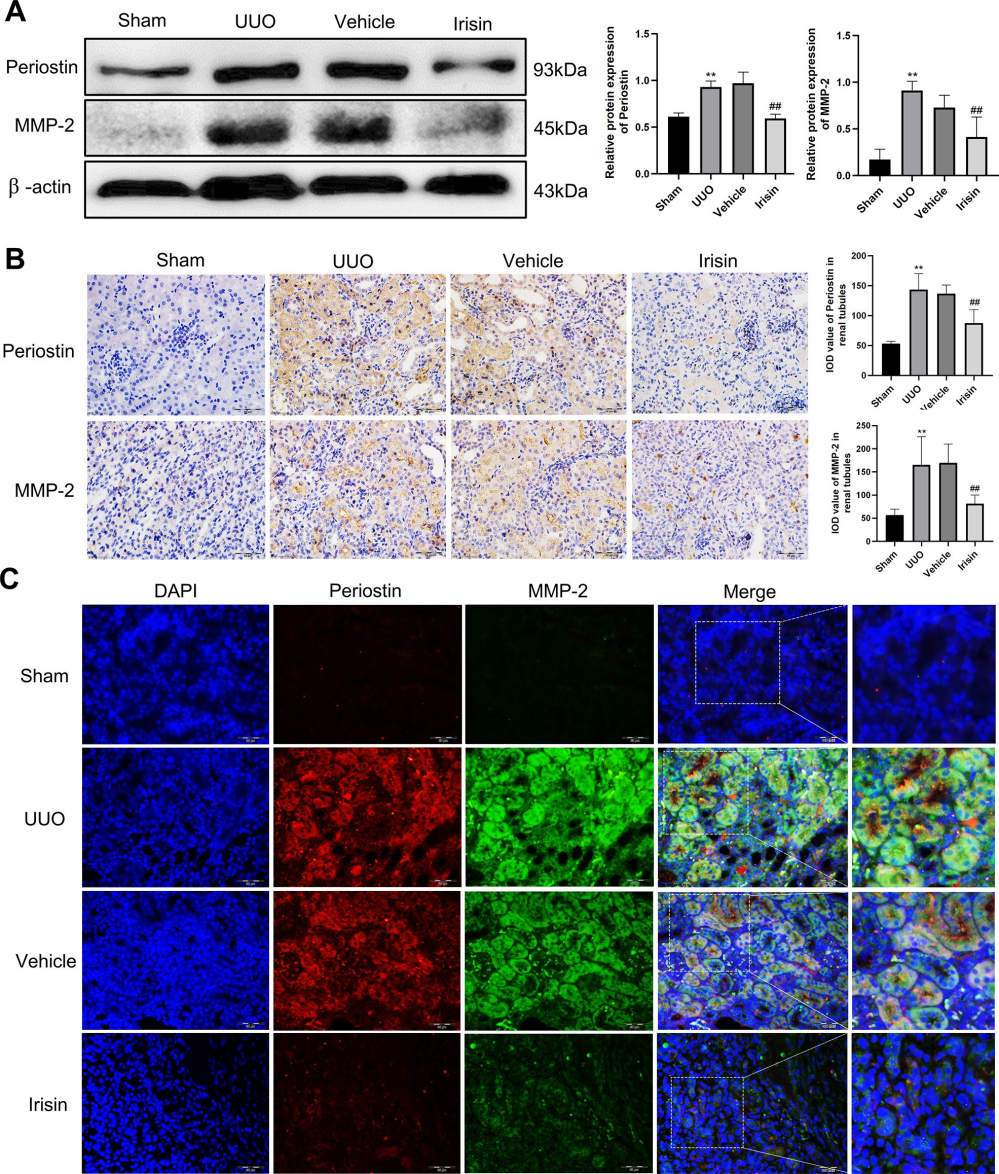
Irisin treatment attenuated UUO-induced the periostin and MMP-2 expression. **A** The representative Western blotting and related quantification of periostin and MMP-2 in renal tissue of mice. **B** The expression of periostin and MMP-2 in renal tissue of Sham, UUO, Vehicle and UUO + irisin mice was detected by IHC. **C** The representative immunofluorescence images of periostin and MMP-2 in renal tissues of Sham, UUO, Vehicle and UUO + irisin mice. All experiments were performed independently at least in triplicate. Values are expressed as means ± SD. *p<0.05 versus Sham group. **p<0.01 versus Sham group. #*p*<0.05 versus Vehicle group. ##*p*<0.01 versus Vehicle group.

In order to further verify the effect of irisin on periostin and MMP-2 expression in renal tissue, the protein levels of periostin and MMP-2 were detected by Western blotting. Furthermore, we found that the protein levels of periostin and MMP-2 were significantly higher in the UUO group than in the irisin treatment and sham groups (**Fig 6A**). As shown in **Fig 6B**, immunohistochemistry (IHC) showed that periostin and MMP-2 positive staining areas were mainly located in the renal tubular epithelial cells of UUO kidneys. Double immunofluorescence staining revealed that periostin and MMP-2 co-localized in UUO kidneys (**Fig 6C**).

### 3.7 Irisin treatment alleviated the renal fibrosis in the UUO kidneys

**Fig 7:**
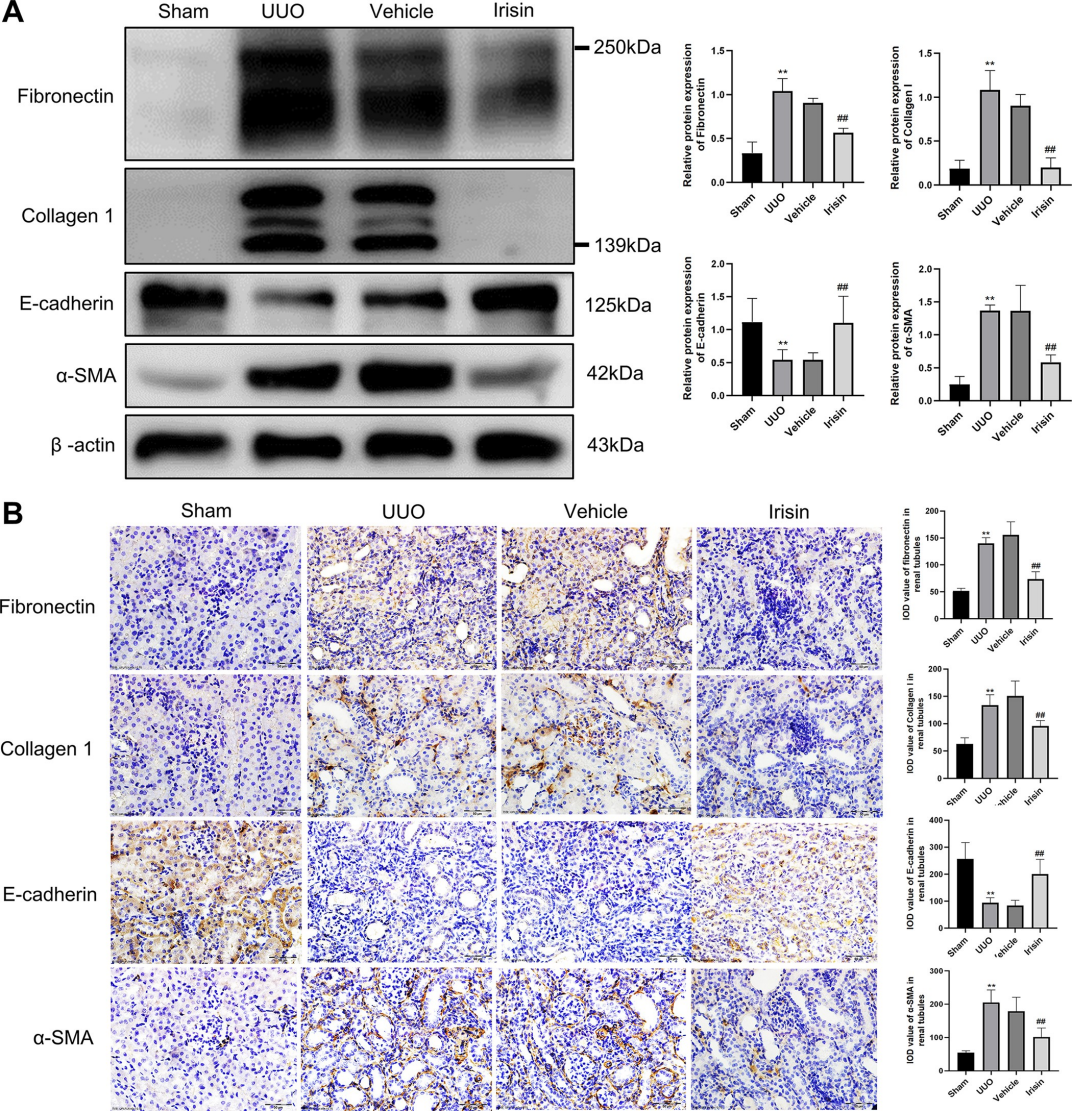
Irisin treatment can reduce renal fibrosis caused by UUO model. **A** The representative Western blotting and related quantification of fibronectin, collagen 1, E-cadherin and α-SMA in renal tissue of mice. **B** The expression of fibronectin, collagen 1, E-cadherin and α-SMA in renal tissue of Sham, UUO, Vehicle and UUO+irisin mice was detected by IHC. All experiments were performed independently at least in triplicate. Values are expressed as means ± SD. *p<0.05 versus Sham group. **p<0.01 versus Sham group. #*p*<0.05 versus Vehicle group. ##*p*<0.01 versus Vehicle group.

To elucidate whether irisin treatment had a protective effect against renal fibrosis, We explored the effect of irisin on ECM deposition and EMT in UUO kidneys. As shown in **Fig 7A**, compared with sham-operated kidneys, the protein expression of fibronectin, collagen 1, and α-SMA was markedly increased in UUO kidneys, and the protein expression of E-cadherin was markedly decreased in UUO kidneys. Unexpectedly, irisin treatment significantly reversed these effects. In addition, the IHC results were consistent with the results of western blotting (**Fig 7B**). These data suggested that irisin treatment effectively attenuated UUO-induced renal injury by inhibiting the periostin/MMP-2 signaling pathway.

## 4. Discussion

Renal fibrosis, characterized by abnormal accumulation of ECM, may manifest as glomerulosclerosis and tubulointerstitial fibrosis (mainly in renal tubules) [15]. However, the molecular mechanisms underlying renal fibrosis remain to be elucidated. Irisin, a muscle factor, is a cleavage product of its precursor FNDC5, and is shed into the extracellular environment and circulation [16]. In recent years, the anti-inflammatory, anti-apoptotic and anti-fibrotic properties of irisin have been widely concerned by the scientific community. These pathogenic processes are often closely related to the development and prognosis of many diseases, such as myocardial infarction [17], kidney disease [16], lung injury [18], liver disease [19] and so on. However, the role of irisin in kidney disease, particularly in UUO models, is not fully understood.

Bioinformatics analysis is a reliable and efficient tool for identifying potential therapeutic targets and diagnostic biomarkers of precision medicine [15]. In this study, we selected differential genes in the UUO model using bioinformatic analysis to further explore the relationship between differential genes and irisin. Bioinformatic analysis was performed based on four GEO datasets to screen for key genes and pathways related to UUO. The GSE121190, GSE36496, GSE42303, and GSE96101 datasets were used to identify differentially expressed genes between the control and UUO mouse kidney tissues. Next, we selected the intersecting genes between these differential genes using a Venn diagram. A total of 656 genes were selected for further analysis. GO functional enrichment analysis showed that these 656 genes were mainly enriched for cell adhesion, membrane, and protein binding. KEGG pathway enrichment analysis showed that differentially expressed genes were mainly enriched in protein digestion and absorption, the PI3K-Akt signaling pathway, phagosome, and AGE-RAGE signaling pathways. PPI network analysis identified six important functional modules and top 20 hub genes. Of the 20 hub genes, periostin and MMP-2 were selected for further investigation.

Periostin is a 90 KD ECM protein originally named osteoblast-specific factor 2, which is highly expressed in bone and tooth tissues. In addition, it is highly expressed in chronic diseases of multiple organs, especially in the kidneys, to participate in the process of inflammation and fibrosis or proliferation [20]. In recent years, TGF-β1 has been reported to induce periostin expression in the inner medullary collecting duct cells in diabetic nephropathy [21] and UUO models [22]. In this study, we found that TGF-β1 increased the expression of periostin in a dose-dependent manner in HK-2 cells, and the expression of periostin was particularly significant after 48 h of incubation.

MMP-2, also known as gelatinase A, is a member of the matrix metalloproteinase family. Normally, human renal mesangial cells and renal tubular epithelial cells produce MMP-2, despite their low levels. However, in renal fibrosis, abnormal activation and interaction of multiple cell signaling pathways, such as the TGF-β/Smad signaling pathway, P38MAPK signaling pathway, and hypoxia, can lead to the upregulation of MMP-2 expression [23]. Recent studies on lung cancer [24] and renal carcinoma [25] have shown that periostin can significantly regulate MMP-2 expression. Our study showed that MMP-2 expression was increased by periostin overexpression. Interestingly, a study by Peng et al. showed that irisin competitively inhibits Smad phosphorylation by binding to TGF-βRII, thereby inhibiting the transmission of the TGF-β signaling pathway [26]. As a result, we think that irisin can prevent renal fibrosis by preventing the TGF-β signaling pathway from causing the production of periostin and MMP-2. In our study, irisin treatment significantly alleviated the expression of periostin and MMP-2 in the kidneys of UUO mice, as well as the expression of periostin and MMP-2 in HK-2 cells induced by TGF-β1.

EMT and ECM accumulation in renal tubular epithelial cells are also involved in UUO- and TGF-β1-induced renal interstitial fibrosis. We investigated the impact of irisin on renal tubular EMT and ECM deposition in this work. Our *in vivo* research revealed that irisin boosted the expression of E-cadherin while decreasing the expression of fibronectin, collagen 1, and α-SMA in the kidney of UUO mice. We also discovered that *in vitro*-cultured human renal tubular epithelial cells exposed to TGF-β1 induced ECM formation, EMT, and inflammatory responses, and that irisin directly blocked these TGF-β1-driven responses. In addition, in cell experiments, we found that the therapeutic effect of irisin seemed to disappear after the overexpression of periostin and MMP-2. These results indicated that the therapeutic effect of irisin may decrease with an increase in fibrosis.

In summary, bioinformatics analysis revealed that periostin and MMP-2 were two significant hub genes in UUO and non-UUO mice. Administration of irisin significantly attenuated TGF-β1-induced ECM, EMT accumulation, and the inflammatory response via the periostin/MMP-2 pathway *in vitro*. However, the therapeutic effect of irisin weakened when periostin and MMP-2 were overexpressed. Moreover, irisin administration significantly attenuated renal EMT and ECM accumulation via the periostin/MMP-2 pathway in the kidneys of UUO mice. These findings indicate that irisin treatment could improve UUO-induced renal interstitial fibrosis and suggest that irisin may be used to treat fibrotic kidney diseases.

## Supporting information

S1 Raw images (DOCX)
