# Supplementary material for: Irisin ameliorates UUO-induced renal interstitial fibrosis through TGF-β1/periostin/MMP-2 signaling pathway
Source: PLoS One. 2024 Jun 13;19(6):e0299389. doi: 10.1371/journal.pone.0299389 (PMC11175535; doi:10.1371/journal.pone.0299389)
Supplement: S1 Raw images — (DOCX) [file pone.0299389.s001.docx]

**Figure2A**

MW

X

Ctrl

1ng/ml

TGF-β1

2.5ng/ml

TGF-β1

5ng/ml

TGF-β1

10ng/ml

TGF-β1

KDa


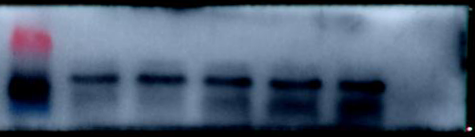


70

100

Periostin(93KDa)


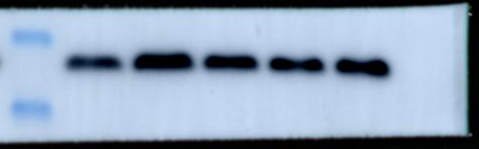


55

40

β-actin(43KDa)


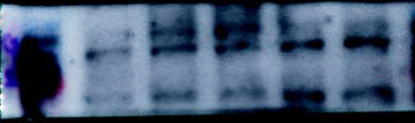


100

70

Periostin(93KDa)


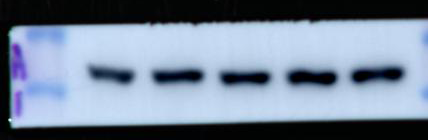


β-actin(43KDa)

40

55


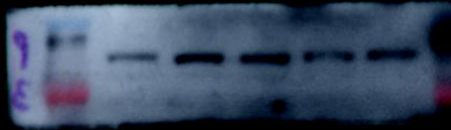


Periostin(93KDa)

70

100


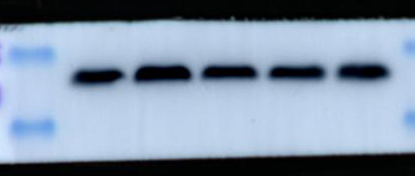


β-actin(43KDa)

40

55

**Figure2B**

X

MW

Ctrl

KDa

72(h)

48

24

12

**
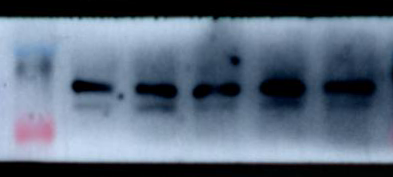
**

Periostin(93KDa)

100

70

**
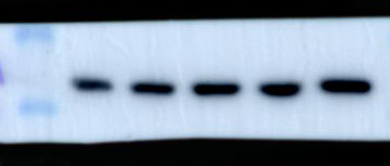
**

β-actin(43KDa)

55

40

**
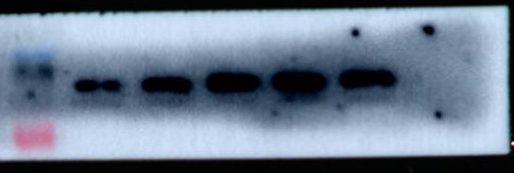
**

Periostin(93KDa)

100

70

**
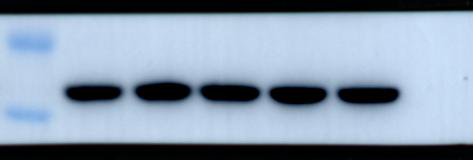
**

β-actin(43KDa)

55

40

**
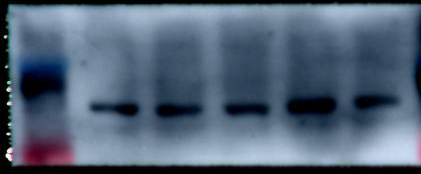
**

Periostin(93KDa)

100

70

**
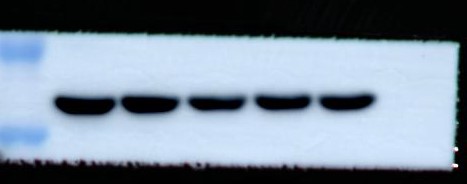
**

β-actin(43KDa)

55

40

X

MW

Ctrl

KDa

**Figure 2C**

TGF-β1

5ng/ml

irsin

10ng/ml

irsin

15ng/ml

irsin

30ng/ml

irsin

**
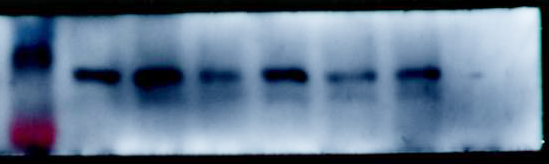
**

Periostin(93KDa)

100

70

**
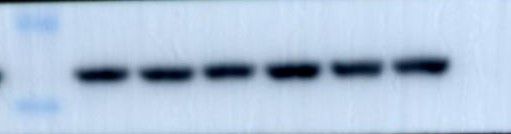
**

β-actin(43KDa)

55

40

**
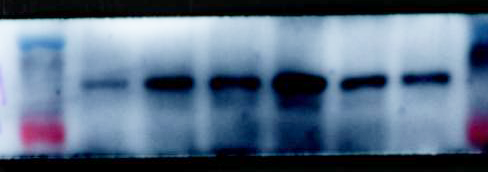
**

Periostin(93KDa)

100

70

**
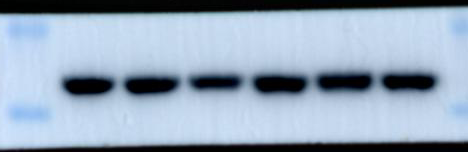
**

β-actin(43KDa)

55

40

**
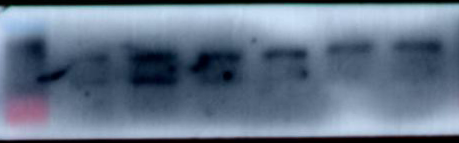
**

Periostin(93KDa)

100

70

**
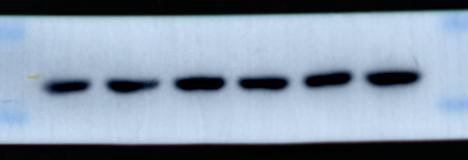
**

55

40

**Figure 2D**

X

MW

β-actin(43KDa)

KDa

Ctrl

TGF-β1

TGF-β1+irisin

**
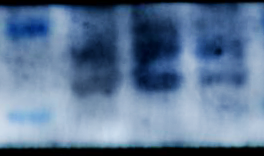
**

55

40

55

40

MMP-2(45KDa)

**
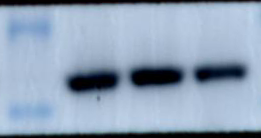
**

β-actin(43KDa)

**
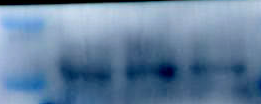
**

55

40

MMP-2(45KDa)

**
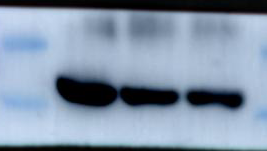
**

55

40

β-actin(43KDa)

**
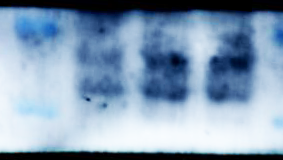
**

55

40

MMP-2(45KDa)

**
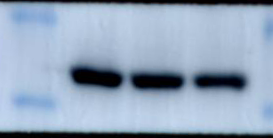
**

β-actin(43KDa)

55

40

**Figure3A**

X

MW

Ctrl

KDa

TGF-β1

TGF-β1+irisin

**
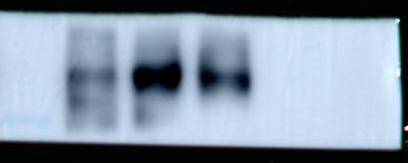
**

Fibronectin(250KDa)

180

**
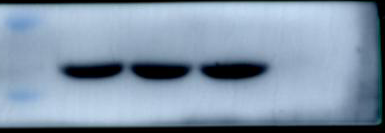
**

β-actin(43KDa)

55

40

**
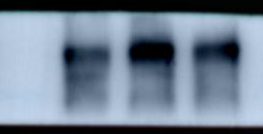
**

Fibronectin(250KDa)

180

**
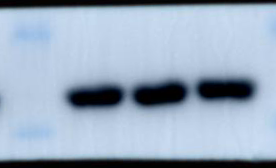
**

β-actin(43KDa)

55

40

**
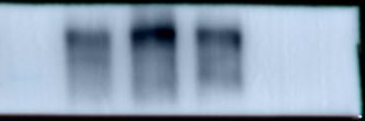
**

Fibronectin(250KDa)

180

**
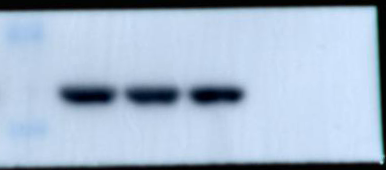
**

β-actin(43KDa)

55

40

**Figure3A**

X

MW

Ctrl

KDa

TGF-β1+irisin

TGF-β1

180

130

100

**
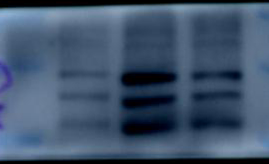
**

Collagen 1(130KDa)

**
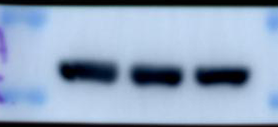
**

β-actin(43KDa)

180

130

100

55

40

**
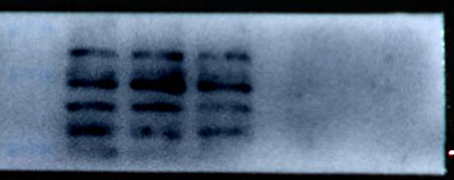
**

Collagen 1(130KDa)

**
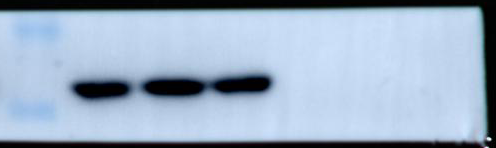
**

β-actin(43KDa)

55

40

180

130

100

**
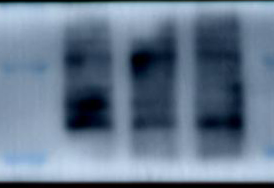
**

Collagen 1(130KDa)

**
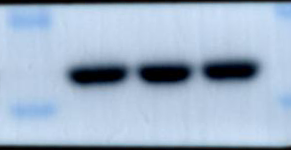
**

β-actin(43KDa)

55

40

**Figure3A**

Ctrl

TGF-β1+irisin

TGF-β1

X

MW

KDa

**
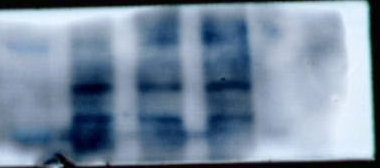
**

E-cadherin(125KDa)

130

100

**
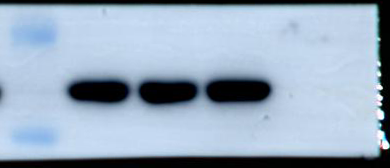
**

β-actin(43KDa)

55

40

**
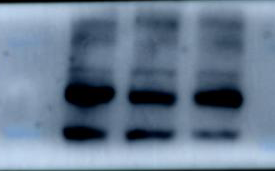
**

E-cadherin(125KDa)

130

100

**
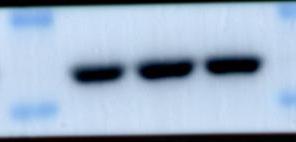
**

β-actin(43KDa)

55

40

**
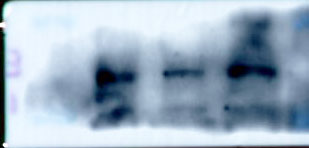
**

E-cadherin(125KDa)

130

100

**
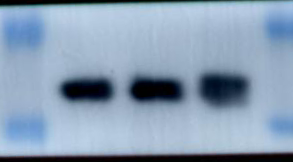
**

β-actin(43KDa)

55

40

**Figure3A**

Ctrl

X

MW

KDa

TGF-β1+irisin

TGF-β1

**
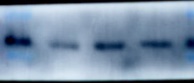
**

α-SMA(42KDa)

55

40

**
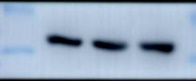
**

β-actin(43KDa)

55

40

**
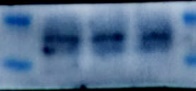
**

α-SMA(42KDa)

55

40

**
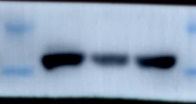
**

β-actin(43KDa)

55

40

**
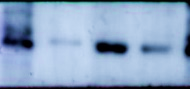
**

α-SMA(42KDa)

55

40

**
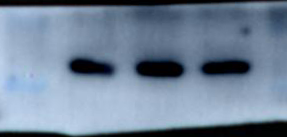
**

55

40

β-actin(43KDa)

**Figure3A**

TGF-β1+irisin

TGF-β1

Ctrl

KDa

X

MW

40

35

**
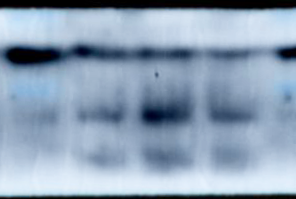

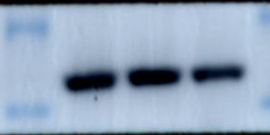
**

TNF-α(26KDa)

β-actin(43KDa)

55

40

**
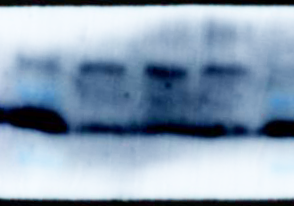
**

X

TNF-α(26KDa)

40

35

**
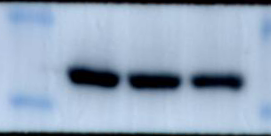
**

40

35

β-actin(43KDa)

55

40

**
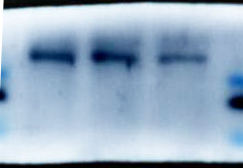
**

TNF-α(26KDa)

**
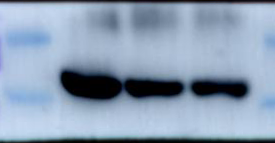
**

β-actin(43KDa)

55

40

**Figure4A**

Ctrl

KDa

X

MW

Pex-2

Periostin

Mmp-2

**
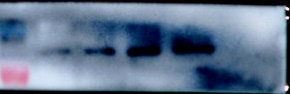

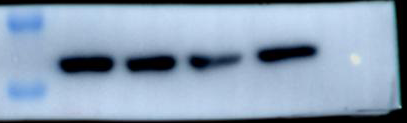
**

Periostin(93KDa)

β-actin(43KDa)

55

40

100

70

**
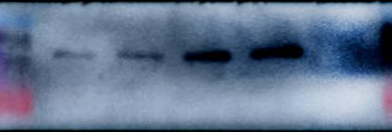
**

Periostin(93KDa)

100

70

**
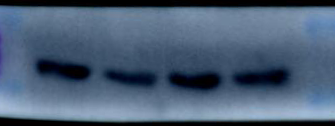
**

β-actin(43KDa)

55

40

**
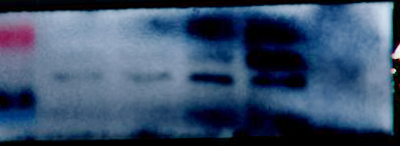

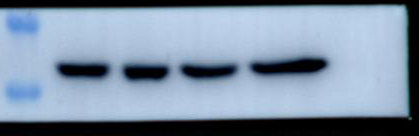
**

55

40

β-actin(43KDa)

Periostin(93KDa)

100

70

**Figure4A**

MMP-2

X

MW

KDa

Ctrl

Pex-2

Periostin

**
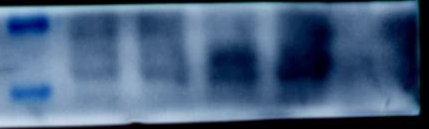
**

MMP-2(45KDa)

55

40

**
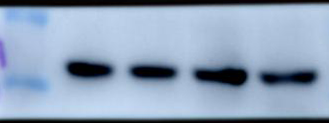
**

β-actin(43KDa)

55

40

**
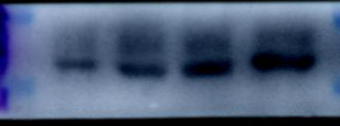
**

MMP-2(45KDa)

55

40

**
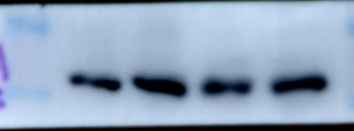

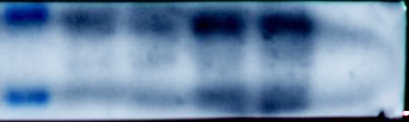
**

β-actin(43KDa)

MMP-2(45KDa)

55

40

55

40

**
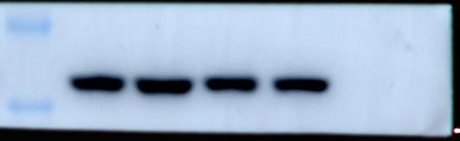
**

β-actin(43KDa)

55

40

**Figure4B**

X

MW

KDa

TGFβ1+irisin

+MMP-2

TGFβ1+irisin

+Periostin

TGFβ1+irisin

**
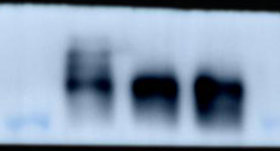

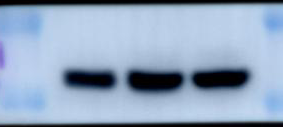
**

β-actin(43KDa)

55

40

180

Fibronectin(250KDa)

**
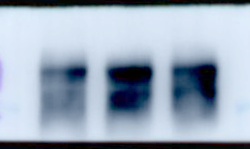

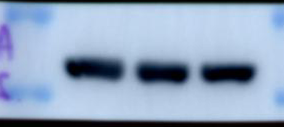
**

Fibronectin(250KDa)

β-actin(43KDa)

55

40

180

**
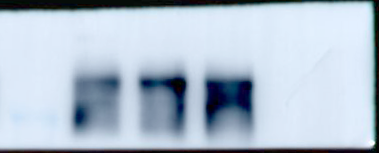
**

Fibronectin(250KDa)

180

**
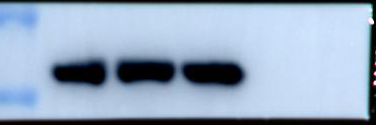
**

β-actin(43KDa)

55

40

**Figure4B**

X

MW

KDa

TGFβ1+irisin

+Periostin

TGFβ1+irisin

+MMP-2

180

130

100

TGFβ1+irisin

**
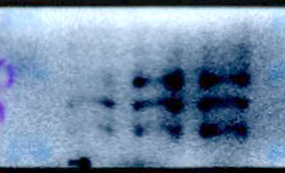
**

Collagen 1(139KDa)

55

40

**
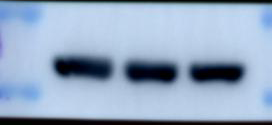

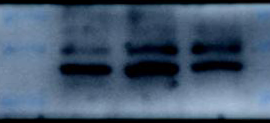

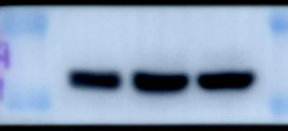
**

β-actin(43KDa)

β-actin(43KDa)

Collagen 1(139KDa)

55

40

180

130

100

**
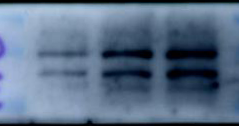

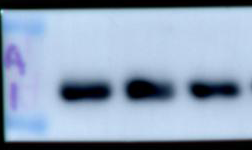
**

β-actin(43KDa)

Collagen 1(139KDa)

55

40

180

130

100

**Figure4B**

X

MW

KDa

TGFβ1+irisin

+Periostin

TGFβ1+irisin

+MMP-2

130

100

TGFβ1+irisin

**
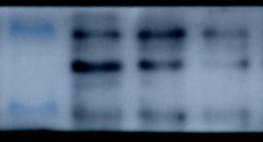
**

E-cadherin 1(125KDa)

**
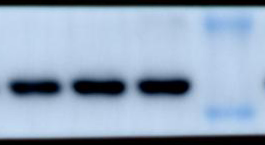

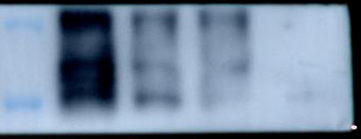
**

β-actin(43KDa)

55

40

E-cadherin 1(125KDa)

130

100

**
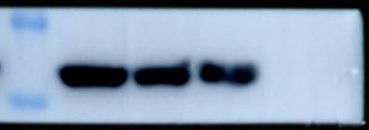
**

130

100

β-actin(43KDa)

55

40

**
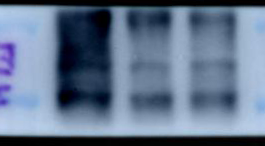
**

E-cadherin 1(125KDa)

**
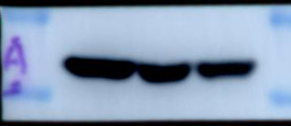
**

β-actin(43KDa)

55

40

**Figure4B**

X

MW

KDa

TGFβ1+irisin

+Periostin

TGFβ1+irisin

TGFβ1+irisin

+MMP-2

**
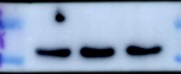
**

α-SMA(42KDa)

55

40

55

40

**
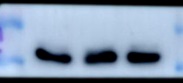

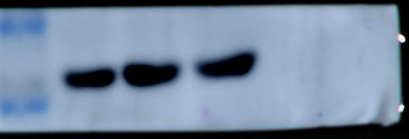

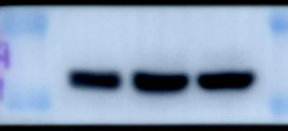

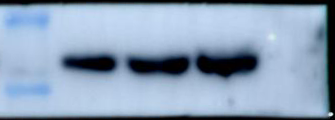

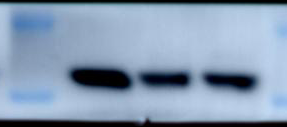
**

β-actin(43KDa)

β-actin(43KDa)

β-actin(43KDa)

α-SMA(42KDa)

α-SMA(42KDa)

55

40

55

40

55

40

55

40

**Figure4B**

TGFβ1+irisin

+Periostin

X

MW

KDa

TGFβ1+irisin

+MMP-2

TGFβ1+irisin

**
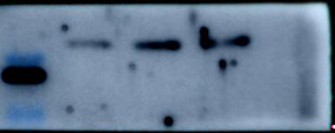

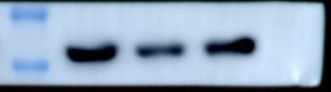
**

TNF-α(26KDa)

β-actin(43KDa)

55

40

25

10

**
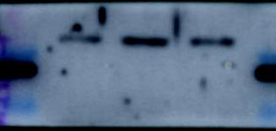

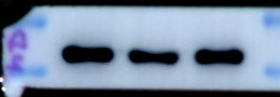
**

β-actin(43KDa)

TNF-α(26KDa)

55

40

25

10

**
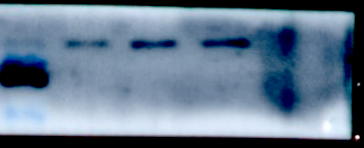

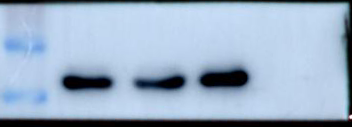
**

55

40

25

10

β-actin(43KDa)

TNF-α(26KDa)

**Figure5A**

X

MW

KDa

Vehicle

UUO

Sham

Irisin

**
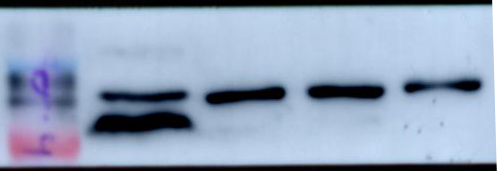
**

Periostin(93KDa)

100

70

**
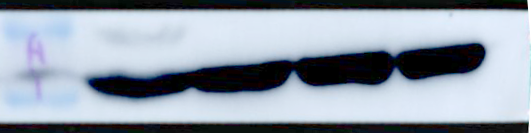
**

β-actin(43KDa)

55

40

**
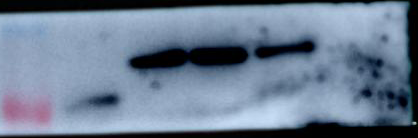
**

Periostin(93KDa)

100

70

**
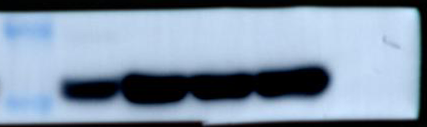
**

β-actin(43KDa)

55

40

Periostin(93KDa)

100

70

β-actin(43KDa)

55

40

**Figure5A**

X

MW

KDa

Sham

UUO

Vehicle

Irisin

MMP-2(45KDa)

55

40

55

40

β-actin(43KDa)

55

40

MMP-2(45KDa)

55

40

β-actin(43KDa)

55

40

MMP-2(45KDa)

55

40

β-actin(43KDa)

**Figure6A**

X

MW

KDa

Sham

UUO

Vehicle

Irisin

180

Fibronectin(250KDa)

55

40

β-actin(43KDa)

180

Fibronectin(250KDa)

55

40

β-actin(43KDa)

180

Fibronectin(250KDa)

β-actin(43KDa)

55

40

**Figure6A**

X

MW

KDa

Sham

UUO

Vehicle

Irisin

Collagen 1(139KDa)

180

130

100

β-actin(43KDa)

β-actin(43KDa)

Collagen 1(139KDa)

55

40

180

130

100

55

40

Collagen 1(139KDa)

180

130

100

β-actin(43KDa)

55

40

**Figure6A**

X

MW

KDa

Sham

UUO

Vehicle

Irisin

E-cadherin(125KDa)

130

100

β-actin(43KDa)

55

40

E-cadherin(125KDa)

130

100

55

40

β-actin(43KDa)

E-cadherin(125KDa)

130

100

β-actin(43KDa)

55

40

E-cadherin(125KDa)

130

100

55

40

β-actin(43KDa)

**Figure6A**

X

MW

KDa

Sham

UUO

Vehicle

Irisin

β-actin(43KDa)

β-actin(43KDa)

α-SMA(42KDa)

55

40

55

40

55

40

α-SMA(42KDa)

55

40

55

40

β-actin(43KDa)

α-SMA(42KDa)

55

40

α-SMA(42KDa)

55

40

β-actin(43KDa)

55

40
